# Frin: An Efficient Method for Representing Genome Evolutionary History

**DOI:** 10.3389/fgene.2019.01261

**Published:** 2019-12-06

**Authors:** Yan Hong, Juan Wang

**Affiliations:** School of Computer Science, Inner Mongolia University, Hohhot, China

**Keywords:** evolution, phylogenetic network, incompatibility degree, frequency, genome

## Abstract

Phylogenetic analysis is important in understanding the process of biological evolution, and phylogenetic trees are used to represent the evolutionary history. Each taxon in a phylogenetic tree has not more than one parent, so phylogenetic trees cannot express the complex evolutionary information implicit in phylogeny. Phylogenetic networks can be used to express genome evolutionary histories. Therefore, it is great significance to research the construction of phylogenetic networks. Cass algorithm is an efficient method for constructing phylogenetic networks because it can construct a much simpler network. However, Cass relies heavily on the order of input data, i.e. different networks can be constructed for the same dataset with different input orders. Based on the frequency and incompatibility degree of taxa, we propose an efficiently improved algorithm of Cass, called as Frin. The experimental results show that the networks constructed by Frin are not only simpler than those constructed by other methods, but Frin can also construct more consistent phylogenetic networks when the treated data have different input orders. Furthermore, the phylogenetic network constructed by Frin is closer to the original information described by phylogenetic trees. Frin has been built as a Java software package and is freely available at https://github.com/wangjuanimu/Frin.

## Introduction

Studying the evolution of species is helpful for humans to reveal biological secrets, prevent, and treat diseases. The purpose of phylogenetic analysis is to reveal the evolutionary relationships between different species or taxa and study the evolution of life on Earth (Huson and Scornavacca, 2011). The evolutionary history is like the growth of trees, and all species can be traced back to a common ancestor. It makes sense to use trees to represent the evolutionary history, in which each node except the root has only one parent. There are a number of reticulate evolutionary events, such as reversal, translocation, and fusion, which have resulted in more than one parent of some taxa in the evolution (Gusfield et al., 2007a; Gusfield et al., 2007b; Kelk and Scornavacca, 2014; Wu, 2010;Van Iersel et al., 2017). Such a complex evolutionary history can be represented by the phylogenetic networks (Doolittle, 1999; Nakhle, 2010; Yu and Nakhleh, 2015; Huber et al., 2018). A network is a generalization of a tree in that it contains nodes with in-degree greater than one (Iersel et al., 2009). Phylogenetic networks are functionally classified into implicit networks and explicit networks (Huson et al., 2007; Huson and Rupp, 2008; Van Iersel et al., 2010). Implicit networks can be used to represent conflicting patterns due to the model misspecification. However, explicit networks can capture reticulate evolutionary events.

In recent years, a lot of work has been developed on the methods for constructing phylogenetic networks (Albrecht 2015; Albrecht et al., 2012; Bordewich et al., 2007; Francis et al., 2018; Gambette et al., 2017; Linz and Semple, 2009; Makarenkov et al., 2006; Mirzaei and Wu, 2016; Jansson and Sung, 2006). Cluster network method uses the network-popping algorithm to construct an implicit network, which can be drawn as a cladogram (Huson and Rupp, 2008). Galled network method uses the seed-growing algorithm to find the solution of RMCS (Restricted Maximum Compatible Subset) problem for input dataset, and then construct phylogenetic network (Huson et al., 2007). The relationships between phylogenetic trees and networks are the basis for the reconstruction and verification of phylogenetic networks. TCP algorithm solved the problem whether or not certain existing phylogenetic trees are displayed in a phylogenetic network (Gunawan et al., 2016; Gunawan et al., 2018). Cass is an efficient method to construct a phylogenetic network for any input trees, and is able to construct much simpler networks than other available methods (Van Iersel et al., 2010). But Cass usually constructs some different networks for the same dataset when it is input as different orders. The phylogenetic network constructed by Cass represents lots of redundant information except for the original information. Both factors considered it is obvious that Cass has poor practical application. Lnetwork improves the Cass by fixing the order of removed taxa in the construction process of phylogenetic networks. It saves the running time for us and reduces the dependence on the input data order (Wang et al., 2013a). BIMLR is also an improved algorithm of Cass by considering incompatibility of taxa in the construction process of phylogenetic network (Wang et al., 2013b). Such methods, including Cass, Lnetwork, and BIMLR, have the significant flexibility that they are not restricted to binary input trees and are not restricted to trees on the same taxa set. In addition, they can construct simpler networks for the same input than other methods, although they are relatively slow. Therefore, The above three methods are efficient and widely used in the construction of phylogenetic networks.

In this paper, we will introduce another improved Cass algorithm, Frin. It constructs phylogenetic networks with phylogenetic trees as input, just like Cass algorithm. Experiments show that Frin is less dependent on the input data order and runs faster than Cass. Moreover, Frin constructs a simpler network than other available methods.

## Preliminaries

### Related Knowledge

Given a set of taxa *X*, a subset of *X*, excluding the empty set and the complete set, is called a cluster. A cluster *C* is non-trivial if it contains more than one element. If two clusters ${C^{\prime }}_{1}$ and ${C^{\prime }}_{2}$ are compatible if either ${C^{\prime }}_{1}\cap {C^{\prime }}_{2}=\phi$ or ${C^{\prime }}_{1}\subset {C^{\prime }}_{2}$ or ${C^{\prime }}_{2}\subset {C^{\prime }}_{1}$. Otherwise, they are incompatible. For a set of cluster *Y* on *X*, *Y* is said to be compatible if any one pair of clusters are compatible. An incompatible cluster set is represented by an incompatible graph *IG*(*Y*) = (*E*, *V*), which consists of a node set and an edge set. The node set consists of all the non-trivial clusters in the *Y* and the edge set consists of edges connecting the incompatible clusters. The set of clusters represented by a rooted phylogenetic tree is compatible; on the contrary, any one compatible cluster set can be constructed into a rooted phylogenetic tree.

Supposed that *N* = (*V*, *E*) is a network on taxa set *X*. *δ*^-^(*v*) represents the in-degree of the node *v*. We introduce a concept used to describe the complexity of a network, which is called reticulation number. Reticulation number of a network is not necessarily equal to the number of reticulate nodes. It is defined as:

$$\sum_{\nu \in V,\;\delta^{-}>0}\left(\delta^{-}(\nu )-1\right)=|E|-|V|+1$$

If each connected component of a network contains reticulation number at most *k*, then we call that it is a *level-k* network. A level-*k* network is called a simple level- < *k* network, which does not contain cut nodes. A node is a cut node if its removal disconnects the graph.

Each phylogenetic tree *T* is uniquely defined by the set of clusters. For a phylogenetic tree, an edge *e* = (*u*, *v*) represents the cluster containing those taxa that are descendants of *v*. Similarly, a phylogenetic network represents clusters in the soft-wired sense or in the hard-wired sense. For each reticulate node of the network *N*, we switch on its one incoming edge and switch off the others, and we called the network *N* represents the cluster *C* in the soft-wired sense if cluster *C* equals the set of all taxa that can be reached from *v*. On the other hand, if cluster *C* equals the set of taxa that are descendants of *v*, we said the edge (*u*, *v*) of a network represents the cluster *C* in the hard-wired sense. In this article, we research the representing in the soft-wired sense, whose pseudocode is shown by Algorithm 1.

**Algorithm 1:** The clusters represented by a network in the soft-wired sense.

**Input:** a phylogenetic network (level-*k*)
**Output:** a cluster set *Y*
**Begin**
1. *Y* = null
2. *i = k-*1; *j*[*k*] = false
3. soft (*N*, *i*, *j*)
4. **for:***v*∈*V*of *N*
5. **if***i* < 0 **then**
6. **if***j* = true **then**
7. switch on the left incoming edge of each reticulate node and switch off the right one
8. **else**
9. switch off the left incoming edge of each reticulate node and switch on the right one
10. **end if**
11. **for:***v*∈*V*of *N*
12. **if** out-degree(*v*) = 0 **then**
13. add a cluster represented by *v* to *Y*
14. **else**
15. add clusters represented by the child of *v* to *Y*
16. **end if**
17. **end for**
18. **else**
19. *j*[*i*] ← true
20. **continue:** soft (*N*, *i*-1, *j*)
21. *j*[*i*] ← false
22. **continue:** soft (*N*, *i*-1, *j*)
23. **end if**
24. **end for**
25. **return** the cluster set *Y*
**End**

Cass, Lnetwork, BIMLR, and Frin all take the set of trees as the input when to construct a phylogenetic network. They first compute all clusters represented by input trees, and then construct a phylogenetic network representing those clusters. Assume that *Y* is the cluster set represented by the input file, *N* is a constructed network. *Y′* is the cluster set represented by the network, which are greater than or equal to the clusters in the *Y*. The clusters in *Y-Y′*are called the redundant clusters. Both the reticulation number and the number of redundant clusters describe the complexity of a network. The best phylogenetic network should contain fewer reticulation numbers and have fewer redundant clusters.

Suppose that *N* is a network on taxa set *X*, *e* = (*u*, *v*) is an edge of *N* with parent node *u* and child node *v*. If each way from the root node to *v* passes through *u*, we called that *u* is the stable ancestor on *v*; otherwise, it is the unstable ancestor. For an edge *e* = (*u*, *v*), let P(*e*) = {*x*∈*X*| *x* is the stable ancestor on *v*}, Q(*e*) = {*x*∈*X* | *x* is the unstable ancestor on *v*}, S(*e*) = {*x*∈*X* | *x* is not a descendant of *v*}. We call {P(*e*), Q(*e*), S(*e*)} the tripartition of *e*. Θ(N) represents all tripartition sets of network *N*. Given two networks *N*_1_ and *N*_2_, tripartition distance between them is computed by $|\Theta (N_{1})\Delta \Theta (N_{2})|/2$, of which Δ is symmetry variation. The tripartition distance measures the topology different between two phylogenetic networks. In this paper, we use the tripartition distance to measure the dissimilarity of the phylogenetic networks.

### Cass Algorithm

We will have a brief description for Cass algorithm in the following. Given a set of clusters *Y* on taxa *X*, Cass algorithm is divided into four steps:

Step 1: Cass works out non-trivial connected component *Y*_1_,…,*Y_p_* of incompatibility graph *IG*(Y). Then, Cass collapses the maximal ST-sets for each non-trivial connected component *Y_i_* and gets $Y_{i}$. Given a taxa set *X* and a subset *S*⊂*X*, each cluster *C*⊂*Y* removes the elements of subset *S*, and the remaining cluster set *Y*′ is called the restriction of *Y* to *S*, denoted by *Y*|*s*. The largest set of ST-set is called the maximal ST-set. Given |S|>1, if *S* is compatible with each cluster of *Y*, and *Y*|*s* are compatible, we called *S* is a strict tree set (ST-set) of *Y*.

Step 2: Cass (*k*) constructs simple level- < *k* networks for each $Y_{i}$, which is crucial step of Cass algorithm. For each non-trivial connected component, Cass(*k*) loops all taxa and removes them from each cluster, and collapses all of the maximal ST-sets for the remaining cluster set. Cass(*k*) repeats above operations *k* times, until the remaining cluster set is compatible to construct phylogenetic trees. The removed taxon is added to the phylogenetic tree as children of reticulate nodes, which becomes a simple level- < *k* network.

Step 3: For each *i*∈{1,…,*p*}, Cass removes all clusters that are in *C_i_*, adds a cluster *X_i_* and each maximal subset *X*⊂*X_i_* that is not separated by *C_i_*. All above set become cluster set $C^{\prime \prime }$. Then Cass constructs a rooted phylogenetic tree *T* for $C^{\prime \prime }$, which is the whole frame of the resulting network.

Step 4: Cass adds all the simple level- < *k* networks constructed in step 2 to the rooted phylogenetic tree *T* by the method of ancestor nodes displacement.

When Cass starts constructing a simple level- < *k* network, it does not know the number of network level. Thus, it first sets *k* = 0 and runs Cass(0),which constructs a simple level- < 0 network. If such a network exists, it outputs the result and halts. Otherwise, Cass continues to sets *k* = *k* + 1, and runs Cass(1), Cass(2),…, Cass(*k*), until the constructed network represents the given clusters sets the soft-wired sense. The process is very time-consuming, because Cass(*k*) loops over all taxa and repeatedly attempts to remove each taxon. The selection of removed taxa is highly uncertain, which makes the algorithm depend heavily on the order of input data, and it also reduces the speed of the construction.

## Method

Given a set of clusters *Y* on taxa set *X*, the frequency of a taxon *x*∈*X* is the number of clusters containing taxon *x*, denoted by *f*(*x*). The number of edges of the graph *IG*(*Y*) is called incompatibility degree of *Y*, denoted by *d*(*Y*). The incompatibility degree of a taxon *x*∈*X*, denoted by *d*(*x*), is the result of subtracting the incompatibility degree of *Y*_|X|{x}_ from that of *Y*, i.e. *d*(*x*) = *d*(*Y*) –*d* (*Y*_|_*_X_*_|{_*_x_*_}_). For example, given incompatible cluster set *Y* = {1, 2}, {2, 3}, we can get taxa frequency *f*(1) = 1, *f*(2) = 2, *f*(3) = 1 and taxa incompatibility degree *d*(1) = 0, *d*(2) = 1, *d*(3) = 0. Moreover, we know that only by removing taxa 2, the remaining clusters are compatible. Frequency and incompatibility degree of taxa contribute a lot to the compatibility of a cluster set, which will affect the construction of phylogenetic networks. The premise of constructing a network is to construct a phylogenetic tree for the compatible cluster set, which is the result by removing some taxa from the originally incompatible set of clusters. The key of Frin method lies in the addition of taxa removal rules, which makes the algorithm select removed taxa more efficiently. Frin chooses the removed taxa based on its frequency and incompatibility degree. Such choices make the remaining cluster set compatible as quickly as possible.

Frin constructs phylogenetic networks in four steps; steps 1, 3, and 4 are the same as Cass algorithm. Frin improves the step 2 of the Cass for the construction of simple level- < *k* networks. Frin first find the non-trivial connected components of the incompatibility graph *IG*(*Y*); next it constructs simple level- < *k* network based on taxa frequency and incompatibility degree; then it constructs a unique phylogenetic trees for compatible clusters; finally it integrates simple level- < *k* networks into the resulting phylogenetic networks. Frin (*k*) constructs a simple level- < *k* network as follows.

For each taxon *x*∈*X*′, Frin(*k*) obtains the frequency and incompatibility degree, and then calculates the weighted value |equ_0013.eps| on the frequency and incompatibility degree, i.e. *s*(*x*) = *p* × *f*(*x*) + *q* × *d*(*x*), where *p* and are *q* weight values of its frequency and incompatibility degree. All taxon *x*∈*X*′ are ordered according to the value of *s*. Frin(*k*) selects the taxon with the maximum *s* as the removed taxa each time, until the remaining cluster set is compatible to construct a phylogenetic tree. Then Frin(*k*) adds all the removed taxa to the tree as the child of reticulate nodes, and gets a resulting network representing all clusters. Here, we set the value of *p* and *q*, 0 < *p* ≤ 1, 0 ≤ *q* <1, *p* + *q* = 1, and step size is 0.1. Then we can get ten groups of *p* and *q* values, for each group of values, Frin(*k*) constructs only one network. To avoid the same network that can be constructed over and over again when it runs, we ignore constructing the same network as before by comparing the taxa removal process. Finally, Frin constructs one or more different networks, and records the network with less reticulation number and redundant clusters as the final phylogenetic network.

In addition, Frin sometimes adds dummy taxa to construct a network. The dummy taxa are removed before outputting the resulting network.

**Figure 1:**
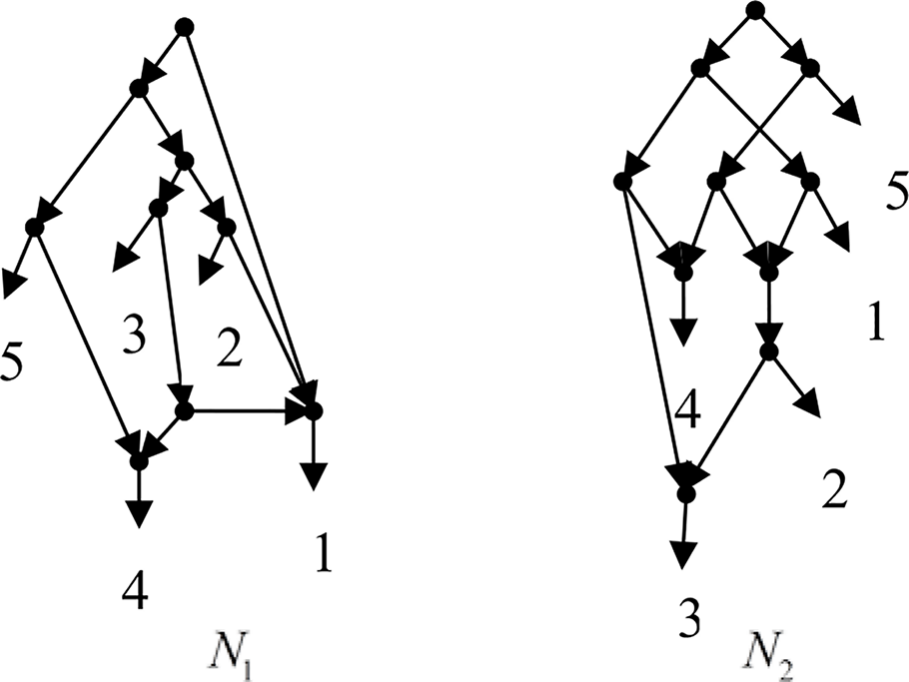
Two networks *N*_1_ and *N*_2_ are constructed by Frin for the cluster set of Example 3.1.

Example 3.1, given taxa set *X* = {1, 2, 3, 4, 5} and the cluster set *Y* = {{1, 2}, {1, 4}, {3, 4}, {1, 3, 4}, {4, 5}, {1, 2, 3, 4}, {2, 3}, {2, 3, 4}, {2, 3, 4, 5}}, Frin constructs two different networks *N*_1_ and *N*_2_ for *Y*, as shown in **Figure 1**. *N*_1_ is a level-3 network with *r* = 3, *c* = 3 and *N*_2_ is a level-3 network with *r* = 3, *c* = 6, where *r* is the reticulation number and *c* is the number of redundant clusters. The two networks have the same reticulation number, and *N*_1_ has fewer redundant clusters than *N*_2_. Therefore, Frin outputs *N*_1_ as the final network. The example shows that Frin can construct several different networks for each input trees due to the coefficients’ uncertainty of the taxa frequency and incompatibility degree. By comparing the number of reticulation nodes and redundant clusters, we select the optimal network from different networks as the output.

**Figure 2:**
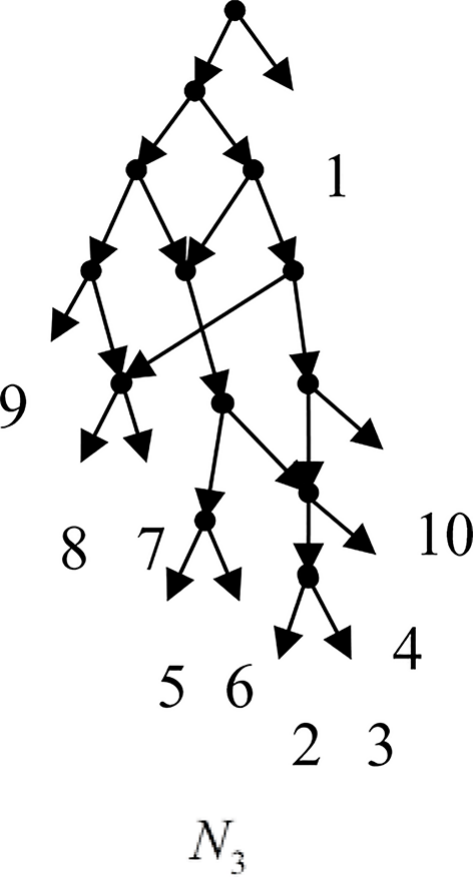
*N*_3_ is the network constructed by Frin for all permutations of input data in Example 3.2.

**Figure 3:**
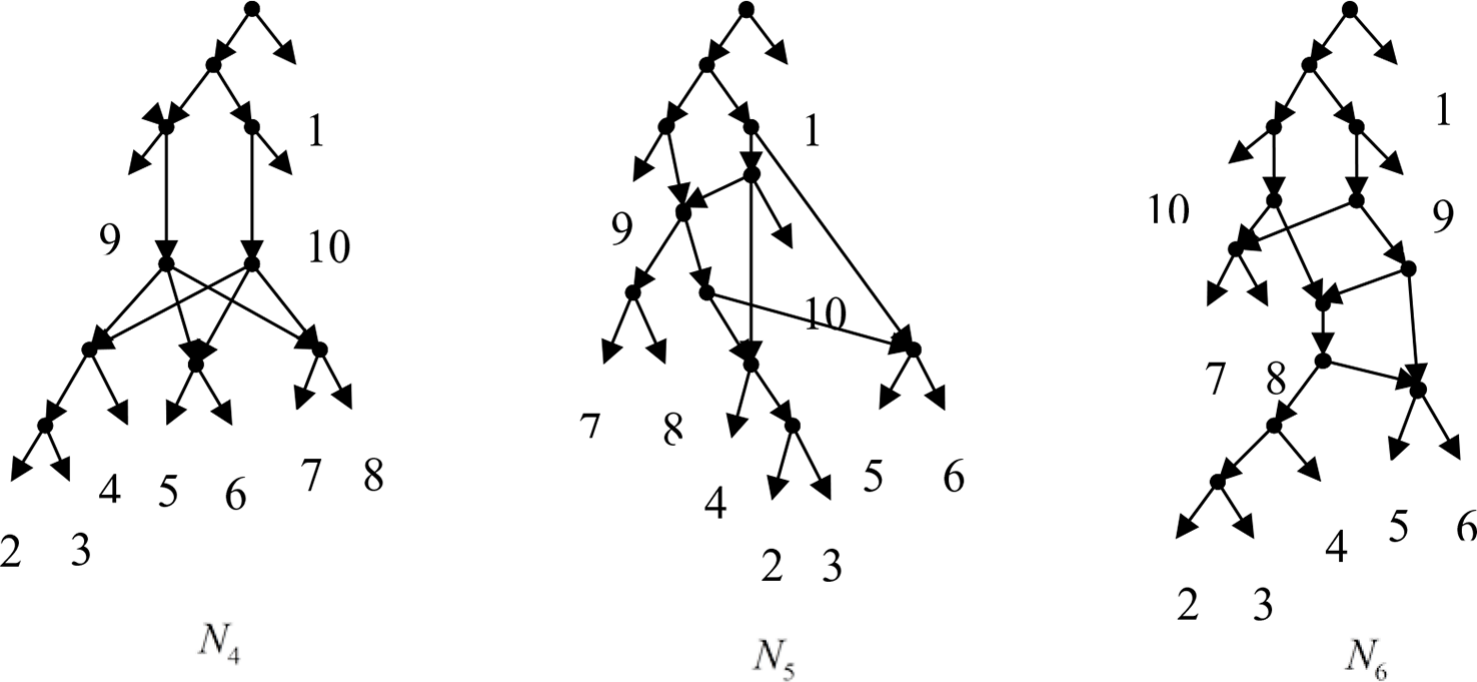
*N*_4_, *N*_5_ and *N*_6_ are the networks constructed by Cass for all permutations of input data in Example 3.2.

**Figure 4:**
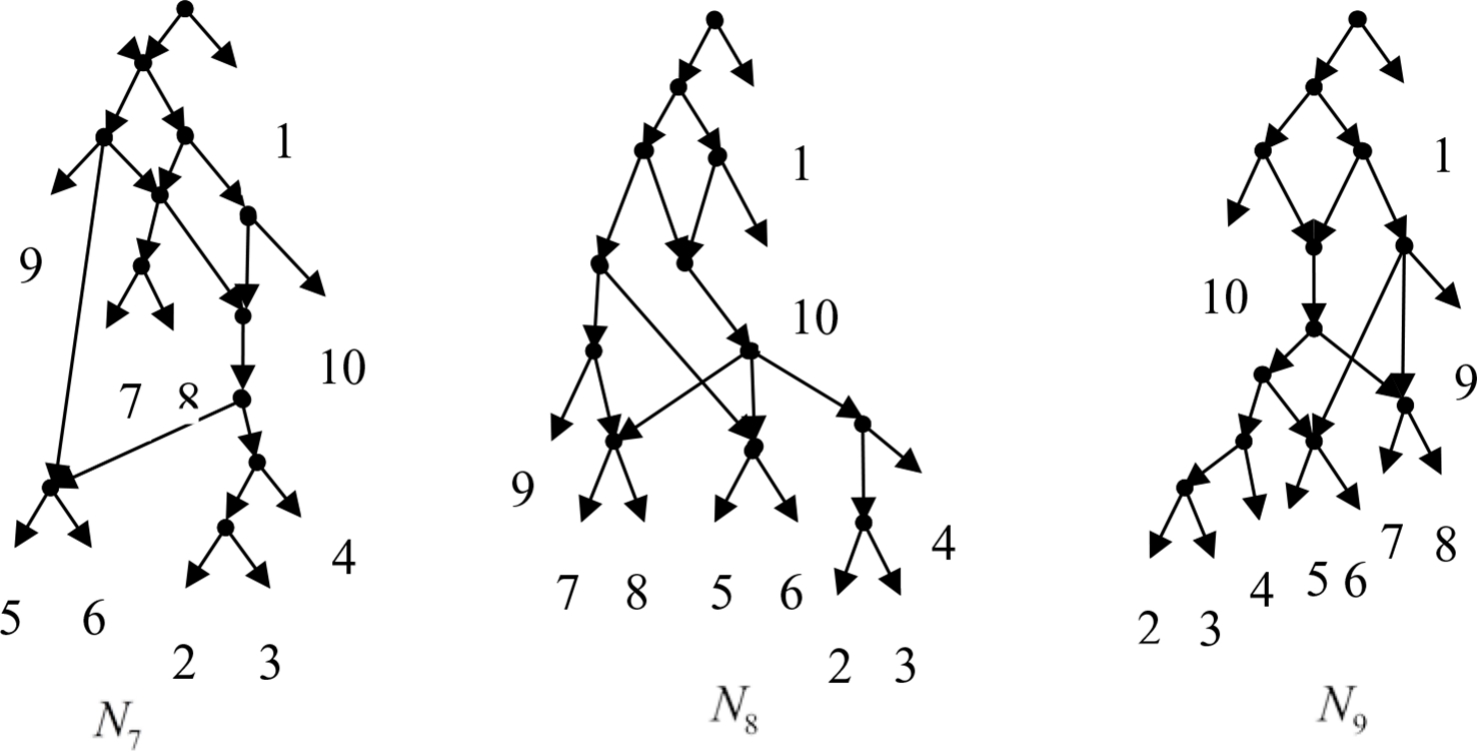
*N*_7_, *N*_8_ and *N*_9_ are the networks constructed by BIMLR for all permutations of input data in Example 3.2.

**Figure 5:**
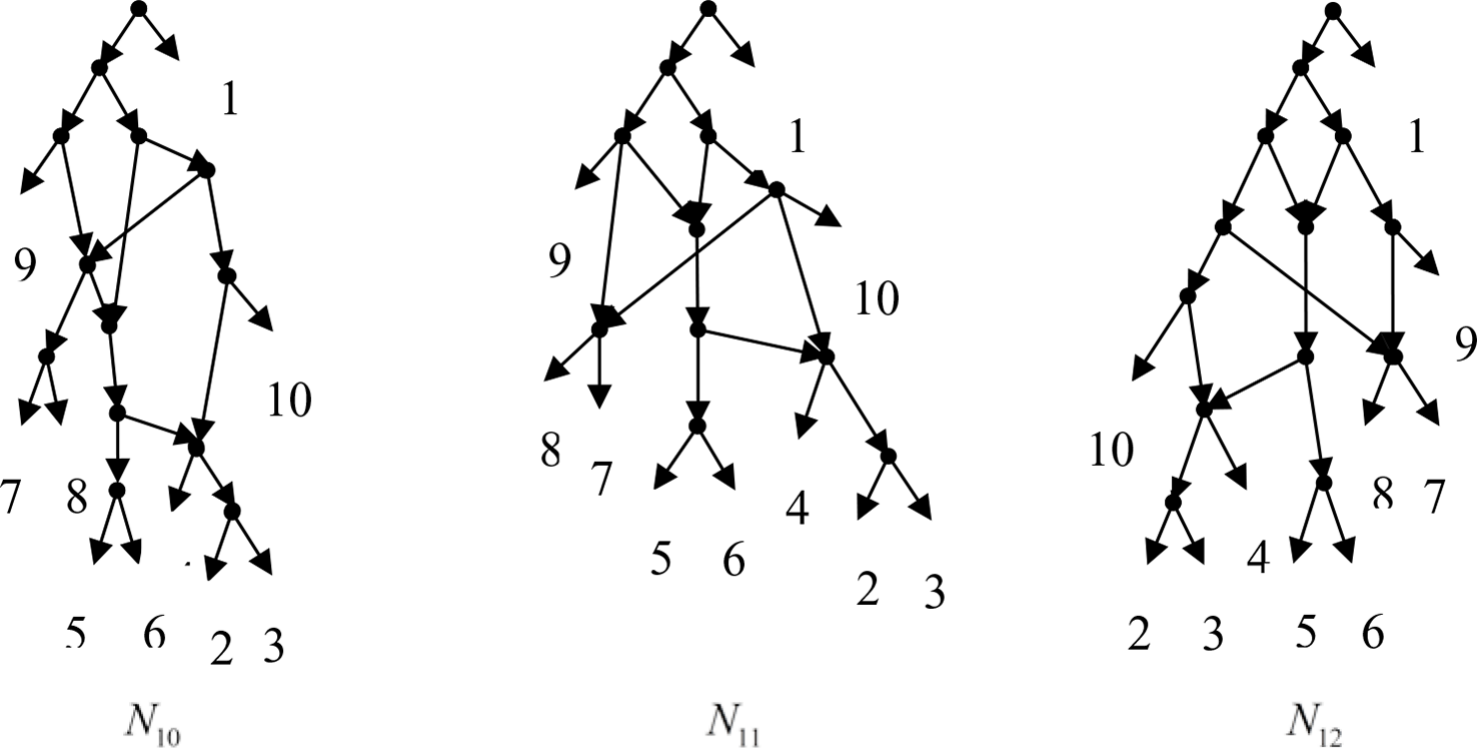
*N*_10_, *N*_11_ and *N*_12_ are the networks constructed by Lnetwork for all permutations of input data in Example 3.2.

Example 3.2, we consider the taxa set *X* = {1, 2, 3, 4, 5, 6, 7, 8, 9, 10} and the cluster set *Y* = {{7, 8, 9}, {2, 3, 4, 7, 8, 10}, {5, 6, 7, 8, 9}, {2, 3, 4, 5, 6, 7, 8, 9}, {2, 3, 4, 5, 6}, {2, 3, 4, 10}, {2, 3, 4, 5, 6, 7, 8, 10}}. We take the cluster set *Y* for example to illustrate that the input data order has different influence degree on Frin, Cass, BIMLR and Lnetwork. Then we need to give all permutations of input data, and construct networks for each permutation. We represent the difference between the resulting networks by tripartition distance. For all permutations of the input data, Frin can construct the same network *N*_3_, as shown in **Figure 2**. Cass constructs three different networks *N*_4_, *N*_5_, and *N*_6_, and the minimum, maximum, and mean tripartition distance between them are 1.5, 2, and 1.67 respectively, as shown in **Figure 3** | *N*_4_, *N*_5_ and *N*_6_ are the networks constructed by Cass for all permutations of input data in Example 3.2. BIMLR constructs three different networks *N*_7_, *N*_8_, and *N*_9_, and the minimum, maximum and mean tripartition distance between them is 1, 3, and 2, as shown in **Figure 4**. Lnetwork also constructs three different networks *N*_10_, *N*_11_, and *N*_12_, and the minimum, maximum, and mean tripartition distance between them is 1, 1.5, and 1.33, as shown in **Figure 5**. The example shows that Frin can construct more consistent networks than other methods for the same data with different input order, i.e. Frin reduces the influence of input data order. The conclusion will be demonstrated by the following section.

## Results

The experiments are performed on a personal computer with an Intel Core i5-4200U, 1.6GHz CPU, and 4GB RAM. All programs are written in Java.

We test the efficiencies of Frin, Cass, Lnetwork, and BIMLR on artificial and the practical dataset, which can be accessed from the website (https://sites.google.com/site/cassalgorithm/data-sets). The results are shown in **Tables 1**–**3**. On the one hand, we use practical data to test the influence of input data order on constructing network (see **Table 1**). On the other hand, we compared the network complexity, i.e. the level; the reticulation number and the redundant cluster number, of four methods on artificial and practical data (see **Table 2** and **3**).

**Table 1:** The results of Frin, Cass, Lnetwork and BIMLR on practical datasets with clusters |*C*| and taxa |*X*| when input order is different.

Data		Firm				Cass				Lnetwork				BIMLR			
|*C*|	|*X*|	n	mean	min	max	n	mean	min	max	n	mean	min	max	n	mean	min	max
35	22	1	0	0	0	2	6.5	6.5	6.5	1	0	0	0	1	0	0	0
25	15	1	0	0	0	2	3	3	3	1	0	0	0	1	0	0	0
22	13	2	1.5	1.5	1.5	2	0.5	0.5	0.5	2	1	1	1	2	1.5	1.5	1.5
27	15	3	3.3	1	5	3	3	3	3	2	1	1	1	2	1	1	1
25	13	1	0	0	0	4	6.3	2	7.5	3	1.2	0.5	1.5	1	0	0	0
22	11	2	5.5	5.5	5.5	3	3	2.5	3.5	1	0	0	0	1	0	0	0
17	10	1	0	0	0	3	2	1.5	2.5	3	1.3	1	1.5	3	2	1	3
13	8	1	0	0	0	4	3.6	1.5	4	2	1	1	1	1	0	0	0
23	11	1	0	0	0	4	5.6	3	7.5	2	1	1	1	2	1	1	1
18	10	1	0	0	0	4	1.5	0.5	3	3	2.5	1.5	3.5	3	1.5	0.5	2.5
22	11	2	0.5	0.5	0.5	3	3.2	1.5	5	1	0	0	0	2	0.5	0.5	0.5
12	11	1	0	0	0	2	3	3	3	1	0	0	0	1	0	0	0
21	10	2	5.5	5.5	5.5	4	3.9	1.5	5.5	2	1.5	1.5	1.5	2	0.5	0.5	0.5
13	7	1	0	0	0	4	3.8	1.5	4	2	1	1	1	1	0	0	0
22	10	3	2.7	2	3.5	2	1.5	1.5	1.5	1	0	0	0	2	0.5	0.5	0.5
21.1	11.8	1.5	1.3	1.1	1.4	3.1	3.4	2.2	4.0	1.8	1.2	1.1	1.4	1.6	0.6	0.4	0.7

**Table 2:** The results of Frin, Cass, Lnetwork and BIMLR on artificial datasets with clusters |*C*| and taxa |*X*|.

Data		Frin				Cass				Lnetwork				BIMLR			
|*C*|	|*X*|	t	k	r	c	t	k	r	c	t	k	r	c	t	k	r	c
86	37	14s	4	9	12	3s	3	8	27	4s	3	8	11	8s	3	8	23
38	20	33s	5	7	11	2s	4	6	25	25s	4	6	15	2s	4	6	25
43	22	1s	3	5	3	1s	2	4	12	1s	3	5	3	1s	3	5	11
72	27	32s	5	7	19	15s	5	7	43	3s	5	7	19	4s	5	7	29
52	22	27s	4	8	12	17s	4	7	33	3s	4	8	15	6s	4	8	15
79	27	3m54s	8	10	80	7m21s	6	8	89	47s	6	8	44	2m40s	8	10	52
38	16	1m44s	6	8	28	15s	5	7	50	4m22s	7	9	36	13s	6	8	25
41	16	2s	4	5	6	1s	4	5	29	1s	4	5	4	1s	4	5	7
12	8	1s	2	2	0	1s	2	2	2	1s	2	2	0	1s	2	2	0
45	20	1m51s	6	7	34	4h4m	6	7	66	35s	6	7	28	17s	6	7	47
22	11	44s	2	3	1	1s	2	3	5	1s	2	3	1	1s	2	3	4
17	10	1s	3	3	4	1s	3	3	8	1s	3	3	4	1s	3	3	7
46	16	6m8s	6	8	10	23s	5	7	34	7s	6	8	15	12s	6	8	22
22	11	41s	4	4	14	2s	4	4	23	3s	4	4	13	2s	5	5	21
22	10	54s	4	4	10	2s	4	4	21	6s	4	4	12	2s	5	5	19
42.3	18.2	1m2s	4.4	6	16	16m51s	3.9	5.5	31	24.9s	4.2	5.8	14.7	15.4s	4.4	6	20.5

**Table 3:** The results of Frin, Cass, Lnetwork and BIMLR on practical datasets with clusters |*C*| and taxa |*X*|.

Data		Frin				Cass				Lnetwork				BIMLR			
|*C*|	|*X*|	t	k	r	c	t	k	r	c	t	k	r	c	t	k	r	c
14	4	1s	3	3	0	1s	3	3	0	1s	3	3	0	1s	3	3	0
30	5	1s	4	4	0	2s	4	4	0	2s	4	4	0	1s	4	4	0
62	6	6s	5	5	0	11s	5	5	0	6s	5	5	0	7s	5	5	0
42	10	1s	4	4	8	5s	4	4	34	1s	4	4	8	1s	4	4	8
39	11	23s	6	6	10	21s	5	5	7	13s	5	5	8	3s	5	5	8
61	11	23s	5	5	11	1m26s	5	5	48	5s	5	5	11	1s	5	5	11
75	30	1s	2	2	19	5s	2	2	122	1s	2	2	19	1s	2	2	19
180	51	8s	2	2	0	40s	2	2	0	4s	2	2	0	1s	2	2	0
70	56	1s	1	4	0	1s	1	4	0	1s	1	4	0	2s	1	4	0
270	76	1m7s	2	2	0	6m22s	2	2	0	12s	2	2	0	24s	2	2	0
404	122	4m1s	2	2	0	1h44m	2	2	0	27s	2	2	0	27s	2	2	0
113.4	34.7	43.7s	3.3	3.5	4.4	10m18s	3.2	3.5	10	6.6s	3.4	3.6	8.5	7.1s	3.2	3.5	4.2

We get all permutations of input order for each data, and then construct networks for each permutation. Since the running time of the experiment is factorial, we choose small-scale data as the input. In order to measure the influence of input data order, we record the number of different resulting networks and compute the tripartition distance between them. We use the tripartition distances to measure the dissimilarity between the networks. The experimental result is shown in **Table 1**. Each dataset consists of cluster number |*C*| and taxa number |*X*|. The table records the number of different networks (n) and mean (mean), minimum (min), maximum (max) values of the tripartition distance, and the last row is the average of the corresponding columns. **Table 1** shows that the number of different networks constructed by Frin is less than other three methods for most data, and the tripartition distance between them is also smaller, especially compared with Cass algorithm. Hence, Frin constructs more consistent networks when the input data orders are different.

We test the complexity of the networks constructed by Frin, Cass, Lnetwork, and BIMLR, including the network level (k), the reticulation number (r) and the redundant cluster number (c), and as well as the running time (t) of those methods in h/m/s. The following tables show the results of experiment on artificial and practical data with the cluster number |*C*| and the taxa number |*X*|. The last row of the tables is the average of the corresponding columns. **Table 2** compares Frin with other three methods in several artificial datasets. It shows that Frin consumes less time for the same input data compared with Cass, and Frin has significantly fewer redundant clusters than Cass and BIMLR. **Table 3** compares the four methods in several practical datasets. It shows that the average reticulation number of Frin is slightly larger than the other methods, but it has fewer redundant clusters than Cass and Lnetwork in most cases. Thus, the network constructed by Frin is simpler than that constructed by other methods in the aspect of redundant clusters, and the execution time of Frin has also been greatly reduced compare with Cass, although it takes longer than the other two methods.

**Figure 6:**
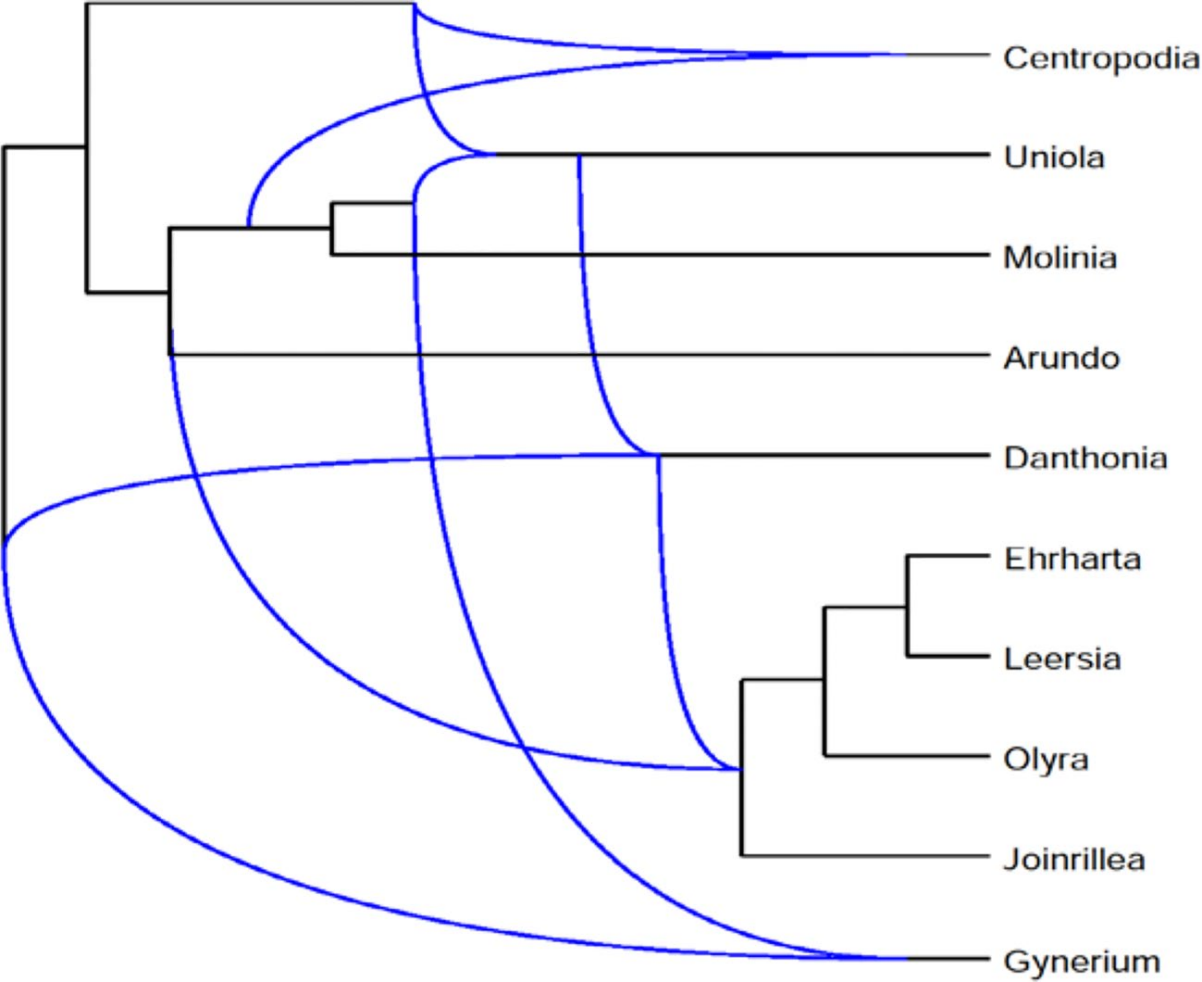
Frin constructs a level-5 network with r = 5, c = 31 for the three gene trees of the *Poaceae* datasets.

We describe the application of Frin to the *Poaceae* dataset and also compare it with other programs. The dataset consists of three phylogenetic trees of the *Poaceae* family, which are based on sequences data for three difference gene loci, petD, ndhB, and rpl2. The gene sequences are downloaded from NCBI database. We do sequence alignment on the obtained sequence using Clustalx, and construct a phylogenetic tree using Phylip. Frin constructs a level-5 network with 10 taxa, 5 reticulations and 31 redundant clusters for the three gene trees of *poaceae* datasets. The resulting network is shown in **Figure 6** using Dendroscope3 (Huson et al., 2007; Vaughan, 2017). For the same input, BIMLR constructs a level-5 network with r = 5, c = 33 and Lnetwork constructs a level-5 network with r = 5, c = 37; while Cass algorithm cannot construct the network in a day. The result shows that the network constructed by Frin is the simplest. It illustrates that the network constructed by Frin which can describe real evolutionary history better than the other methods.

## Conclusion

In this paper, we propose an efficient method called Frin to construct phylogenetic networks. In the process of construction, Frin considers the two factors that affect the compatibility of a cluster set, which are the frequency and incompatibility degree of taxa, respectively. Frin can construct several different networks, and select the simplest network from them as the resulting network. The experimental results show that Frin is an improved method. First, Frin can construct less different networks when the input data order is different than the other methods. Second, the networks constructed by Frin have less the number of redundant clusters than the other methods in the case of the level and the reticulation number of the networks not are increasing. Both facts indicate that Frin can better describe the biological evolutionary history.

## Data Availability Statement

The datasets generated for this study can be found in Github (https://github.com/wangjuanimu/Frin). The artificial and the practical datasets can be accessed from the Cass website (https://sites.google.com/site/cassalgorithm/data231sets).

## Author Contributions

YH proposed the method and designed the experiments. YH and JW wrote the paper.

## Funding

The work was supported by National Natural Science Foundation of China under Grant No. 61661040.

## Conflict of Interest

The authors declare that the research was conducted in the absence of any commercial or financial relationships that could be construed as a potential conflict of interest.
